# 6-(4-Amino­phen­yl)-2-eth­oxy-4-(2-thien­yl)nicotinonitrile

**DOI:** 10.1107/S160053681002369X

**Published:** 2010-06-26

**Authors:** Hoong-Kun Fun, Suchada Chantrapromma, Thawanrat Kobkeatthawin, Mahesh Padaki, Arun M. Isloor

**Affiliations:** a X-ray Crystallography Unit, School of Physics, Universiti Sains Malaysia, 11800 USM, Penang, Malaysia; b Crystal Materials Research Unit, Department of Chemistry, Faculty of Science, Prince of Songkla University, Hat-Yai, Songkhla 90112, Thailand; c Department of Chemistry, National Institute of Technology–Karnataka, Surathkal, Mangalore 575 025, India

## Abstract

In the title nicotinonitrile derivative, C_18_H_15_N_3_OS, the central pyridyl ring makes dihedral angles of 25.22 (10) and 24.80 (16)° with the 4-amino­phenyl and thio­phene rings, respectively. The thio­phene ring is disordered over two orientations by rotation around the C(thio­phene)—C(pyridine) bond; the occupancies are 0.858 (2) and 0.142 (2). The eth­oxy group is slightly twisted from the attached pyridyl ring [C—O—C—C torsion angle = 171.13 (16)°]. In the crystal structure, mol­ecules are linked by N—H⋯N hydrogen bonds into chains along [010]. These chains are stacked along the *a* axis. C—H⋯π weak inter­actions involving the thio­phene ring are observed.

## Related literature

For reference bond-length data, see: Allen *et al.* (1987 ▶). For the synthesis and applications of nicotinonitrile derivatives, see: Amr & Abdulla (2006 ▶); Borgna *et al.* (1993 ▶); Fun *et al.* (2009 ▶); Goda *et al.* (2004 ▶); Kamal *et al.* (2007 ▶); Malinka *et al.* (1998 ▶). For related structures, see: Chantrapromma *et al.* (2009 ▶, 2010 ▶); Fun *et al.* (2009 ▶). For the stability of the temperature controller used in the data collection, see Cosier & Glazer (1986 ▶).
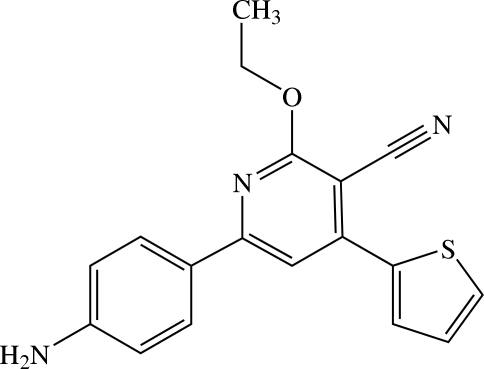


## Experimental

### 

#### Crystal data

C_18_H_15_N_3_OS
*M* *_r_* = 321.38
Orthorhombic, 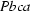

*a* = 7.0751 (12) Å
*b* = 20.843 (4) Å
*c* = 20.983 (4) Å
*V* = 3094.3 (9) Å^3^
*Z* = 8
Mo *K*α radiation
μ = 0.22 mm^−1^
*T* = 100 K
0.35 × 0.11 × 0.04 mm

#### Data collection

Bruker APEXII DUO CCD area-detector diffractometer
Absorption correction: multi-scan (*SADABS*; Bruker, 2009 ▶) *T*_min_ = 0.928, *T*_max_ = 0.992
34805 measured reflections
3045 independent reflections
2188 reflections with *I* > 2σ(*I*)
*R*_int_ = 0.092

#### Refinement

*R*[*F* ^2^ > 2σ(*F* ^2^)] = 0.038
*wR*(*F* ^2^) = 0.085
*S* = 1.05
3045 reflections
233 parameters
88 restraints
H atoms treated by a mixture of independent and constrained refinement
Δρ_max_ = 0.21 e Å^−3^
Δρ_min_ = −0.32 e Å^−3^

### 

Data collection: *APEX2* (Bruker, 2009 ▶); cell refinement: *SAINT* (Bruker, 2009 ▶); data reduction: *SAINT*; program(s) used to solve structure: *SHELXTL* (Sheldrick, 2008 ▶); program(s) used to refine structure: *SHELXTL*; molecular graphics: *SHELXTL*; software used to prepare material for publication: *SHELXTL* and *PLATON* (Spek, 2009 ▶).

## Supplementary Material

Crystal structure: contains datablocks global, I. DOI: 10.1107/S160053681002369X/wn2394sup1.cif

Structure factors: contains datablocks I. DOI: 10.1107/S160053681002369X/wn2394Isup2.hkl

Additional supplementary materials:  crystallographic information; 3D view; checkCIF report

## Figures and Tables

**Table 1:** Hydrogen-bond geometry (Å, °) *Cg*1 is the centroid of the major disorder component of the thio­phene ring.

*D*—H⋯*A*	*D*—H	H⋯*A*	*D*⋯*A*	*D*—H⋯*A*
N2—H1*N*2⋯N3^i^	0.92 (2)	2.29 (2)	3.197 (3)	168.2 (19)
C3—H3*A*⋯*Cg*1^ii^	0.93	2.93	3.566 (6)	127
C12—H12*A*⋯*Cg*1^iii^	0.93	2.78	3.430 (3)	128

Symmetry codes: (i) 
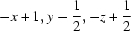
; (ii) 
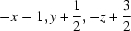
; (iii) 
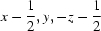
.

**Fig. 1.**
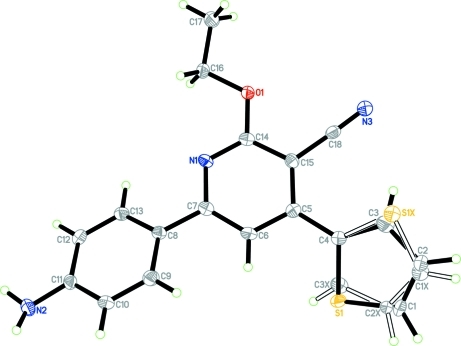
The molecular structure of the title compound, showing 50% probability displacement ellipsoids and the atom-numbering scheme. Hydrogen atoms are shown as spheres of arbitrary radius. The major and minor components of the disorder are shown by shaded and open bonds, respectively.

**Fig. 2.**
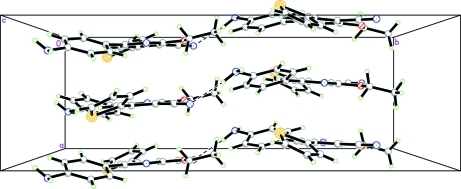
The crystal packing of the title compound, viewed approximately along the c axis, showing chains along [010]. Hydrogen bonds are shown as dashed lines. Only the major disorder components are shown.

## supplementary crystallographic information

### Comment

Heterocyclic compounds containing the pyridine ring are reported to possess a diverse range of biological activities such as antimicrobial, antitumor and anti-inflammatory (Amr & Abdulla, 2006; Borgna *et al.*, 1993; Goda *et al.*, 2004; Kamal *et al.*, 2007; Malinka *et al.*, 1998) properties. Our research is aimed at the synthesis and preliminary biological (*in vitro*) and pharmacological (*in vivo*) screening, together with enzyme inhibitory activity, of the nicotinonitrile derivatives. The title compound, which is a substituted pyridine compound, was synthesized by cyclization of our previous chalcone derivative (Fun *et al.*, 2009) and malononitrile.

The molecule of the title compound, C_18_H_15_N_3_OS, is not planar (Fig. 1). The central pyridyl ring is inclined to the 4-aminophenyl and thiophene rings with dihedral angles of 25.22 (10)° and 24.80 (16)°, respectively. The thiophene ring is disordered over two orientations by rotation around the C4—C5 bond, with occupancies of 0.858 (2) and 0.142 (1). The ethoxy group is twisted slightly from the attached pyridyl ring, as indicated by the torsion angles C14—O1—C16—C17 = 171.13 (16)° and C16—O1—C14—C15 = 179.01 (16) °. The bond distances agree with the literature values (Allen *et al.*, 1987) and are comparable with those for related structures (Chantrapromma *et al.*, 2009; 2010).

In the crystal structure, (Fig. 2), the molecules are linked by weak intermolecular N2—H1N2···N3 hydrogen bond (Table 1) into chains along [010]. These chains are stacked along the *a* axis. The crystal structure is further stabilized by C—H···π interactions (Table 1); *Cg*_1_ is the centroid of the C1–C4/S1 ring (major disorder component).

### Experimental

(2*E*)-1-(4-Aminophenyl)-3-(2-thienyl)prop-2-en-1-one (0.34 g, 0.0015 mole) which was synthesized according to a previous procedure (Fun *et al.*, 2009) was added with continuous stirring to a freshly prepared sodium alkoxide solution (0.0014 mole of sodium in 100 ml of ethanol). Malononitrile (1.30 g, 0.02 mol) was then added with continuous stirring at room temperature until the precipitate separated out. The resulting solid was filtered (yield 68%). Yellow plate-shaped single crystals of the title compound suitable for *X*-ray structure determination were recrystalized from ethanol by the slow evaporation of the solvent at room temperature over several days. Mp. 470–471 K.

### Refinement

The amino H atoms were located in difference maps and refined isotropically. The remaining H atoms were positioned geometrically and allowed to ride on their parent atoms, with d(C—H) = 0.93 Å for aromatic, 0.97 for CH_2_ and 0.96 Å for CH_3_ atoms. The *U*_iso_ values were constrained to be 1.5*U*_eq_ of the carrier atom for methyl H atoms and 1.2*U*_eq_ for the remaining carbon-bound H atoms. A rotating group model was used for the methyl groups. Atoms S1, C1, C2, C3 of the thiophene ring are disordered over two positions by rotation about the C4—C5 bond; the occupancies are 0.858 (2) and 0.142 (2).

### Crystal data

**Table:**

C_18_H_15_N_3_OS	*D*_x_ = 1.380 Mg m^−^^3^
*M**_r_* = 321.38	Melting point = 470–471 K
Orthorhombic, *P**b**c**a*	Mo *K*α radiation, λ = 0.71073 Å
Hall symbol: -P 2ac 2ab	Cell parameters from 3045 reflections
*a* = 7.0751 (12) Å	θ = 1.9–26.0°
*b* = 20.843 (4) Å	µ = 0.22 mm^−^^1^
*c* = 20.983 (4) Å	*T* = 100 K
*V* = 3094.3 (9) Å^3^	Plate, yellow
*Z* = 8	0.35 × 0.11 × 0.04 mm
*F*(000) = 1344	

### Data collection

**Table:**

Bruker APEXII DUO CCD area-detector diffractometer	3045 independent reflections
Radiation source: sealed tube	2188 reflections with *I* > 2σ(*I*)
graphite	*R*_int_ = 0.092
φ and ω scans	θ_max_ = 26.0°, θ_min_ = 1.9°
Absorption correction: multi-scan (*SADABS*; Bruker, 2009)	*h* = −8→8
*T*_min_ = 0.928, *T*_max_ = 0.992	*k* = −25→24
34805 measured reflections	*l* = −25→25

### Refinement

**Table:**

Refinement on *F*^2^	Primary atom site location: structure-invariant direct methods
Least-squares matrix: full	Secondary atom site location: difference Fourier map
*R*[*F*^2^ > 2σ(*F*^2^)] = 0.038	Hydrogen site location: inferred from neighbouring sites
*wR*(*F*^2^) = 0.085	H atoms treated by a mixture of independent and constrained refinement
*S* = 1.05	*w* = 1/[σ^2^(*F*_o_^2^) + (0.0278*P*)^2^ + 1.8001*P*] where *P* = (*F*_o_^2^ + 2*F*_c_^2^)/3
3045 reflections	(Δ/σ)_max_ < 0.001
233 parameters	Δρ_max_ = 0.21 e Å^−^^3^
88 restraints	Δρ_min_ = −0.32 e Å^−^^3^

### Special details

**Table:**

Experimental. The crystal was placed in the cold stream of an Oxford Cryosystems Cobra open-flow nitrogen cryostat (Cosier & Glazer, 1986) operating at 100.0 (1) K.
Geometry. All e.s.d.'s (except the e.s.d. in the dihedral angle between two l.s. planes) are estimated using the full covariance matrix. The cell e.s.d.'s are taken into account individually in the estimation of e.s.d.'s in distances, angles and torsion angles; correlations between e.s.d.'s in cell parameters are only used when they are defined by crystal symmetry. An approximate (isotropic) treatment of cell e.s.d.'s is used for estimating e.s.d.'s involving l.s. planes.
Refinement. Refinement of *F*^2^ against ALL reflections. The weighted *R*-factor *wR* and goodness of fit *S* are based on *F*^2^, conventional *R*-factors *R* are based on *F*, with *F* set to zero for negative *F*^2^. The threshold expression of *F*^2^ > σ(*F*^2^) is used only for calculating *R*-factors(gt) *etc*. and is not relevant to the choice of reflections for refinement. *R*-factors based on *F*^2^ are statistically about twice as large as those based on *F*, and *R*- factors based on ALL data will be even larger.

### Fractional atomic coordinates and isotropic or equivalent isotropic displacement parameters (Å^2^)

**Table:**

	*x*	*y*	*z*	*U*_iso_*/*U*_eq_	Occ. (<1)
O1	0.56053 (19)	0.37231 (6)	0.25979 (6)	0.0190 (3)	
N1	0.5847 (2)	0.26630 (7)	0.22980 (7)	0.0163 (4)	
N2	0.6671 (3)	0.01670 (9)	0.05279 (9)	0.0245 (4)	
N3	0.5866 (2)	0.39328 (8)	0.41791 (8)	0.0245 (4)	
C4	0.6016 (3)	0.21000 (9)	0.42677 (9)	0.0167 (4)	
S1	0.68410 (11)	0.13404 (3)	0.44617 (3)	0.01858 (19)	0.8583 (19)
C1	0.6115 (5)	0.14171 (15)	0.52389 (16)	0.0192 (7)	0.8583 (19)
H1A	0.6270	0.1101	0.5547	0.023*	0.8583 (19)
C2	0.5284 (8)	0.1996 (2)	0.53499 (16)	0.0168 (8)	0.8583 (19)
H2A	0.4807	0.2124	0.5743	0.020*	0.8583 (19)
C3	0.5240 (8)	0.2374 (2)	0.4797 (2)	0.0231 (10)	0.8583 (19)
H3A	0.4718	0.2783	0.4791	0.028*	0.8583 (19)
S1X	0.5077 (14)	0.2515 (4)	0.4850 (4)	0.0200 (18)*	0.1417 (19)
C1X	0.560 (5)	0.1904 (12)	0.5376 (10)	0.021 (8)*	0.1417 (19)
H1XA	0.5349	0.1919	0.5810	0.025*	0.1417 (19)
C2X	0.643 (3)	0.1399 (10)	0.5079 (8)	0.012 (5)*	0.1417 (19)
H2XA	0.6719	0.1011	0.5274	0.014*	0.1417 (19)
C3X	0.679 (3)	0.1544 (8)	0.4425 (9)	0.0231 (10)	0.14
H3XA	0.7470	0.1284	0.4149	0.028*	0.1417 (19)
C5	0.6017 (3)	0.23136 (9)	0.36009 (9)	0.0163 (4)	
C6	0.6082 (3)	0.18612 (9)	0.31136 (9)	0.0176 (4)	
H6A	0.6197	0.1429	0.3217	0.021*	
C7	0.5978 (3)	0.20381 (9)	0.24772 (9)	0.0164 (4)	
C8	0.6032 (3)	0.15594 (9)	0.19636 (9)	0.0158 (4)	
C9	0.5488 (3)	0.09205 (9)	0.20666 (9)	0.0203 (4)	
H9A	0.5016	0.0800	0.2463	0.024*	
C10	0.5641 (3)	0.04666 (9)	0.15893 (9)	0.0201 (4)	
H10A	0.5253	0.0047	0.1666	0.024*	
C11	0.6372 (3)	0.06310 (9)	0.09920 (9)	0.0178 (4)	
C12	0.6864 (3)	0.12704 (9)	0.08808 (9)	0.0181 (4)	
H12A	0.7319	0.1392	0.0483	0.022*	
C13	0.6680 (3)	0.17231 (9)	0.13553 (9)	0.0171 (4)	
H13A	0.6995	0.2147	0.1269	0.021*	
C14	0.5779 (3)	0.30963 (9)	0.27536 (9)	0.0168 (4)	
C15	0.5874 (3)	0.29610 (9)	0.34132 (9)	0.0163 (4)	
C16	0.5537 (3)	0.38737 (9)	0.19226 (9)	0.0197 (4)	
H16A	0.4594	0.3611	0.1712	0.024*	
H16B	0.6754	0.3791	0.1727	0.024*	
C17	0.5035 (3)	0.45707 (9)	0.18628 (10)	0.0248 (5)	
H17A	0.5012	0.4689	0.1421	0.037*	
H17B	0.5961	0.4825	0.2082	0.037*	
H17C	0.3813	0.4644	0.2047	0.037*	
C18	0.5861 (3)	0.34927 (9)	0.38470 (9)	0.0181 (4)	
H1N2	0.586 (3)	−0.0177 (12)	0.0555 (11)	0.039 (7)*	
H2N2	0.678 (3)	0.0308 (10)	0.0137 (11)	0.029 (6)*	

### Atomic displacement parameters (Å^2^)

**Table:**

	*U*^11^	*U*^22^	*U*^33^	*U*^12^	*U*^13^	*U*^23^
O1	0.0293 (7)	0.0135 (7)	0.0143 (7)	0.0002 (6)	0.0006 (6)	0.0000 (6)
N1	0.0175 (8)	0.0123 (9)	0.0191 (9)	−0.0004 (7)	0.0020 (7)	−0.0014 (7)
N2	0.0355 (10)	0.0196 (10)	0.0184 (9)	−0.0040 (8)	−0.0002 (9)	−0.0040 (8)
N3	0.0352 (10)	0.0189 (10)	0.0195 (9)	0.0024 (8)	0.0004 (8)	0.0006 (8)
C4	0.0162 (9)	0.0156 (10)	0.0182 (10)	0.0001 (8)	−0.0005 (8)	0.0003 (8)
S1	0.0220 (3)	0.0159 (4)	0.0179 (3)	0.0049 (3)	0.0012 (2)	0.0032 (3)
C1	0.0239 (16)	0.0225 (16)	0.0113 (15)	0.0003 (12)	0.0022 (13)	0.0024 (13)
C2	0.015 (2)	0.0202 (19)	0.0150 (15)	0.0005 (13)	0.0000 (11)	0.0003 (10)
C3	0.0245 (19)	0.019 (2)	0.025 (2)	−0.0002 (17)	0.0011 (13)	−0.0016 (17)
C3X	0.0245 (19)	0.019 (2)	0.025 (2)	−0.0002 (17)	0.0011 (13)	−0.0016 (17)
C5	0.0132 (9)	0.0181 (10)	0.0177 (10)	0.0004 (8)	0.0000 (8)	0.0014 (8)
C6	0.0178 (9)	0.0123 (10)	0.0227 (11)	0.0007 (8)	0.0019 (8)	0.0005 (8)
C7	0.0136 (9)	0.0154 (10)	0.0202 (10)	−0.0013 (8)	0.0019 (8)	−0.0003 (9)
C8	0.0157 (9)	0.0144 (10)	0.0172 (10)	0.0012 (8)	0.0009 (8)	0.0002 (8)
C9	0.0222 (10)	0.0195 (11)	0.0192 (10)	−0.0001 (9)	0.0026 (8)	0.0016 (9)
C10	0.0247 (10)	0.0138 (10)	0.0217 (11)	−0.0015 (8)	−0.0007 (9)	0.0009 (8)
C11	0.0179 (10)	0.0191 (10)	0.0163 (10)	0.0024 (8)	−0.0038 (8)	−0.0024 (8)
C12	0.0171 (9)	0.0217 (11)	0.0156 (10)	−0.0017 (9)	−0.0013 (8)	0.0029 (8)
C13	0.0161 (9)	0.0160 (10)	0.0193 (10)	−0.0005 (8)	−0.0008 (8)	0.0015 (8)
C14	0.0152 (9)	0.0145 (11)	0.0207 (10)	0.0007 (8)	0.0010 (8)	0.0009 (8)
C15	0.0146 (9)	0.0169 (10)	0.0174 (10)	0.0003 (8)	0.0020 (8)	−0.0015 (8)
C16	0.0265 (10)	0.0186 (11)	0.0141 (10)	−0.0010 (9)	0.0004 (8)	0.0002 (8)
C17	0.0347 (12)	0.0198 (11)	0.0200 (11)	0.0012 (9)	0.0033 (9)	0.0007 (9)
C18	0.0197 (10)	0.0173 (11)	0.0173 (10)	0.0019 (8)	0.0009 (8)	0.0048 (9)

### Geometric parameters (Å, °)

**Table:**

O1—C14	1.352 (2)	C3X—H3XA	0.9300
O1—C16	1.452 (2)	C5—C6	1.392 (3)
N1—C14	1.316 (2)	C5—C15	1.409 (3)
N1—C7	1.359 (2)	C6—C7	1.387 (3)
N2—C11	1.389 (2)	C6—H6A	0.9300
N2—H1N2	0.92 (2)	C7—C8	1.469 (3)
N2—H2N2	0.87 (2)	C8—C13	1.398 (3)
N3—C18	1.152 (2)	C8—C9	1.403 (3)
C4—C3X	1.323 (14)	C9—C10	1.382 (3)
C4—C3	1.364 (5)	C9—H9A	0.9300
C4—C5	1.468 (3)	C10—C11	1.399 (3)
C4—S1X	1.638 (8)	C10—H10A	0.9300
C4—S1	1.736 (2)	C11—C12	1.397 (3)
S1—C1	1.717 (3)	C12—C13	1.378 (3)
C1—C2	1.361 (4)	C12—H12A	0.9300
C1—H1A	0.9300	C13—H13A	0.9300
C2—C3	1.404 (6)	C14—C15	1.414 (3)
C2—H2A	0.9300	C15—C18	1.434 (3)
C3—H3A	0.9300	C16—C17	1.501 (3)
S1X—C1X	1.725 (17)	C16—H16A	0.9700
C1X—C2X	1.355 (16)	C16—H16B	0.9700
C1X—H1XA	0.9300	C17—H17A	0.9600
C2X—C3X	1.427 (17)	C17—H17B	0.9600
C2X—H2XA	0.9300	C17—H17C	0.9600
			
C14—O1—C16	116.59 (14)	N1—C7—C8	116.73 (17)
C14—N1—C7	117.34 (16)	C6—C7—C8	121.62 (17)
C11—N2—H1N2	113.9 (15)	C13—C8—C9	117.52 (17)
C11—N2—H2N2	116.0 (14)	C13—C8—C7	120.83 (17)
H1N2—N2—H2N2	112 (2)	C9—C8—C7	121.63 (17)
C3X—C4—C3	109.2 (9)	C10—C9—C8	121.11 (18)
C3X—C4—C5	120.2 (8)	C10—C9—H9A	119.4
C3—C4—C5	130.5 (3)	C8—C9—H9A	119.4
C3X—C4—S1X	116.3 (8)	C9—C10—C11	120.71 (18)
C5—C4—S1X	123.5 (3)	C9—C10—H10A	119.6
C3—C4—S1	109.1 (2)	C11—C10—H10A	119.6
C5—C4—S1	119.99 (14)	N2—C11—C12	120.63 (18)
S1X—C4—S1	116.3 (3)	N2—C11—C10	120.94 (18)
C1—S1—C4	92.13 (12)	C12—C11—C10	118.39 (17)
C2—C1—S1	112.0 (3)	C13—C12—C11	120.61 (18)
C2—C1—H1A	124.0	C13—C12—H12A	119.7
S1—C1—H1A	124.0	C11—C12—H12A	119.7
C1—C2—C3	111.5 (3)	C12—C13—C8	121.57 (18)
C1—C2—H2A	124.3	C12—C13—H13A	119.2
C3—C2—H2A	124.3	C8—C13—H13A	119.2
C4—C3—C2	115.4 (4)	N1—C14—O1	119.39 (17)
C4—C3—H3A	122.3	N1—C14—C15	124.92 (17)
C2—C3—H3A	122.3	O1—C14—C15	115.69 (16)
C4—S1X—C1X	90.0 (8)	C5—C15—C14	117.90 (17)
C2X—C1X—S1X	111.9 (15)	C5—C15—C18	124.26 (17)
C2X—C1X—H1XA	124.1	C14—C15—C18	117.83 (17)
S1X—C1X—H1XA	124.1	O1—C16—C17	107.39 (15)
C1X—C2X—C3X	110.7 (16)	O1—C16—H16A	110.2
C1X—C2X—H2XA	124.6	C17—C16—H16A	110.2
C3X—C2X—H2XA	124.6	O1—C16—H16B	110.2
C4—C3X—C2X	110.6 (14)	C17—C16—H16B	110.2
C4—C3X—H3XA	124.7	H16A—C16—H16B	108.5
C2X—C3X—H3XA	124.7	C16—C17—H17A	109.5
C6—C5—C15	116.48 (17)	C16—C17—H17B	109.5
C6—C5—C4	119.64 (17)	H17A—C17—H17B	109.5
C15—C5—C4	123.82 (17)	C16—C17—H17C	109.5
C7—C6—C5	121.69 (18)	H17A—C17—H17C	109.5
C7—C6—H6A	119.2	H17B—C17—H17C	109.5
C5—C6—H6A	119.2	N3—C18—C15	177.8 (2)
N1—C7—C6	121.64 (17)		
			
C3X—C4—S1—C1	93 (10)	C14—N1—C7—C6	−1.5 (3)
C3—C4—S1—C1	0.1 (3)	C14—N1—C7—C8	179.37 (16)
C5—C4—S1—C1	−172.59 (18)	C5—C6—C7—N1	1.4 (3)
S1X—C4—S1—C1	1.6 (4)	C5—C6—C7—C8	−179.56 (17)
C4—S1—C1—C2	−0.2 (3)	N1—C7—C8—C13	25.1 (3)
S1—C1—C2—C3	0.2 (5)	C6—C7—C8—C13	−154.02 (19)
C3X—C4—C3—C2	−5.3 (12)	N1—C7—C8—C9	−156.59 (17)
C5—C4—C3—C2	171.7 (3)	C6—C7—C8—C9	24.3 (3)
S1X—C4—C3—C2	−170 (5)	C13—C8—C9—C10	1.9 (3)
S1—C4—C3—C2	−0.1 (5)	C7—C8—C9—C10	−176.49 (18)
C1—C2—C3—C4	−0.1 (7)	C8—C9—C10—C11	1.0 (3)
C3X—C4—S1X—C1X	−3(2)	C9—C10—C11—N2	174.78 (18)
C3—C4—S1X—C1X	14 (4)	C9—C10—C11—C12	−2.9 (3)
C5—C4—S1X—C1X	176.6 (14)	N2—C11—C12—C13	−175.82 (17)
S1—C4—S1X—C1X	2.6 (15)	C10—C11—C12—C13	1.8 (3)
C4—S1X—C1X—C2X	−2(3)	C11—C12—C13—C8	1.1 (3)
S1X—C1X—C2X—C3X	6(4)	C9—C8—C13—C12	−2.9 (3)
C3—C4—C3X—C2X	4(2)	C7—C8—C13—C12	175.46 (17)
C5—C4—C3X—C2X	−173.0 (13)	C7—N1—C14—O1	−178.52 (15)
S1X—C4—C3X—C2X	6(2)	C7—N1—C14—C15	1.5 (3)
S1—C4—C3X—C2X	−85 (10)	C16—O1—C14—N1	−1.0 (2)
C1X—C2X—C3X—C4	−8(3)	C16—O1—C14—C15	179.01 (16)
C3X—C4—C5—C6	26.4 (13)	C6—C5—C15—C14	0.9 (3)
C3—C4—C5—C6	−150.2 (4)	C4—C5—C15—C14	−176.28 (17)
S1X—C4—C5—C6	−153.1 (5)	C6—C5—C15—C18	−177.58 (17)
S1—C4—C5—C6	20.7 (2)	C4—C5—C15—C18	5.2 (3)
C3X—C4—C5—C15	−156.5 (12)	N1—C14—C15—C5	−1.2 (3)
C3—C4—C5—C15	26.9 (4)	O1—C14—C15—C5	178.77 (16)
S1X—C4—C5—C15	24.1 (5)	N1—C14—C15—C18	177.37 (17)
S1—C4—C5—C15	−162.13 (15)	O1—C14—C15—C18	−2.6 (3)
C15—C5—C6—C7	−1.0 (3)	C14—O1—C16—C17	171.13 (16)
C4—C5—C6—C7	176.29 (17)		

### Hydrogen-bond geometry (Å, °)

**Table:**

Cg1 is the centroid of the major disorder component of the thiophene ring.

**Table:**

*D*—H···*A*	*D*—H	H···*A*	*D*···*A*	*D*—H···*A*
N2—H1N2···N3^i^	0.92 (2)	2.29 (2)	3.197 (3)	168.2 (19)
C3—H3A···Cg1^ii^	0.93	2.93	3.566 (6)	127
C12—H12A···Cg1^iii^	0.93	2.78	3.430 (3)	128

Symmetry codes: (i) −*x*+1, *y*−1/2, −*z*+1/2; (ii) −*x*−1, *y*+1/2, −*z*+3/2; (iii) *x*−1/2, *y*, −*z*−1/2.
